# A sliding stem in revision total knee arthroplasty provides stability and reduces stress shielding: An RSA study using impaction bone grafting in synthetic femora

**DOI:** 10.3109/17453674.2010.483991

**Published:** 2010-05-21

**Authors:** Huub J Meijerink, Corné J M van Loon, Maarten C de Waal Malefijt, Albert van Kampen, Nico Verdonschot

**Affiliations:** ^1^Department of Orthopaedics, Radboud University Nijmegen Medical Centre, Nijmegen; ^2^Department of Orthopaedics, Rijnstate Hospital, Arnhem; ^3^Laboratory for Biomechanical Engineering, University of Twente, Enschedethe Netherlands

## Abstract

**Background and purpose:**

In the reconstruction of unicondylar femoral bone defects with morselized bone grafts in revision total knee arthroplasty, a stem extension appears to be critical to obtain adequate mechanical stability. Whether stability is still assured by this reconstruction technique in bicondylar defects has not been assessed. The disadvantage of relatively stiff stem extensions is that bone resorption is promoted due to stress shielding. We therefore designed a stem that would permit axial sliding movements of the articulating part relative to the intramedullary stem.

**Methods:**

This stem was used in the reconstruction with impaction bone grafting (IBG) of 5 synthetic distal femora with a bicondylar defect. A cyclically axial load was applied to the prosthetic condyles to assess the stability of the reconstruction. Radiostereometry was used to determine the migrations of the femoral component with a rigidly connected stem, a sliding stem, and no stem extension.

**Results:**

We found a stable reconstruction of the bicondylar femoral defects with IBG in the case of a rigidly connected stem. After disconnecting the stem, the femoral component showed substantially more migrations. With a sliding stem, rotational migrations were similar to those of a rigidly connected stem. However, the sliding stem allowed proximal migration of the condylar component, thereby compressing the IBG.

**Interpretation:**

The presence of a functional stem extension is important for the stability of a bicondylar reconstruction. A sliding stem provides adequate stability, while stress shielding is reduced because compressive contact forces are still transmitted to the distal femoral bone.

## Introduction

In revision total knee arthroplasty (TKA) the distal femoral bone stock may be compromised as a result of stress shielding, polyethylene wear, or loosening of the femoral component (Cadambi et al. 1994, Liu et al. 1995, Petersen et al. 1995, Robinson et al. 1995, van Lenthe et al. 1997, van Loon et al. 1999a). Smaller unicondylar defects may be treated with morselized bone grafts and mechanical tests have indicated that a stable situation can be created in unicondylar femoral bone defect cases (van Loon et al. 1999b). However, femoral bone defects encountered in TKA are frequently bicondylar and lack cortical support. In these cases, the use of a femoral stem extension has been suggested to provide adequate postoperative stability and to protect bone grafts from failing by fracture, disintegration, or non-union (Ghazavi et al. 1997, Engh and Ammeen 1999). In the reconstruction of larger unicondylar femoral bone defects, a stem extension appears to be important in order to obtain adequate mechanical stability under loaded conditions (van Loon et al. 2000c). However, whether the stability is still assured by this reconstruction technique (bone grafts in combination with a stem extension) in cases with severe bicondylar bony defects has not been assessed.

With the limited clinical experience of impacted bone grafting (IBG) in revision knee surgery as reported in the literature, a lack of stability has emerged as a main concern (Bradley 2000, van Loon et al. 2000a, Heyligers et al. 2001, Toms et al. 2005). As a result, long, rigid stem extensions have been used to maximize the stability of the reconstruction. Although these stems may ensure greater initial stability, the disadvantage of femoral components extended with relatively stiff stems is that long-term bone resorption is promoted due to stress shielding (Bourne and Finlay 1986, van Lenthe et al. 1997, 2002). Moreover, strain on the impacted bone graft may contribute to bony incorporation (Board et al. 2008). Thus, there appears to be incompatibility caused by the fact that on the one hand direct postoperative stability is improved, whereas on the other hand long-term bone quality is jeopardized by a stem extension. The challenge is therefore to develop a system that creates the same stability as with a stem extension, yet does not contribute to stress shielding.

Finite-element (FE) models of TKAs predict less bone resorption when the femoral reconstruction is less rigid with a thinner stem (instead of a thick pressfit stem) or a fully unbonded prosthesis-cement interface (van Lenthe et al. 2002). Thus, we developed a relatively thin intramedullary stem that permitted axial sliding movements of the articulating part relative to the intramedullary stem. The hypothesis behind the design was that compressive contact forces would be directly transmitted to the distal femoral bone, whereas adequate stability would be provided by the sliding intramedullary stem. A prototype was made of this new knee revision design and it was applied to the reconstruction of uncontained bicondylar femoral bone defects with IBG.

We analyzed the stability of the reconstruction of uncontained bicondylar femoral bone defects in TKA with IBG and a thin stem extension. In addition, we determined the differences in stability between a rigidly connected stem, a sliding stem, and no stem extension. The stability was analyzed by radiostereometric analysis (RSA).

## Materials and methods

### Designs and operative technique

Distal synthetic femora were used and bicondylar defects were created that were reconstructed with impaction bone grafting. Subsequently, a stemmed femoral component was implanted and tested for mechanical stability. Using a custom-made connection between the stem and the condylar part of the prosthesis, it was possible to assess the stability of the reconstruction using a fixed stem, a sliding stem, and a disconnected stem in sequential order for one reconstructed specimen.

**Figure 1.**
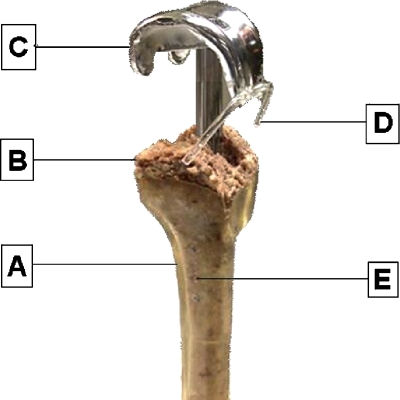
The reconstructed distal femur just before implantation of the stemmed femoral component. A. Synthetic distal femur in upside-down position. B. Reconstructed condyle with IBG. C. Femoral component with a thin cement layer. D. Connector with tantalum pellets glued at the flange for RSA measurements. E. Tantalum pellet inserted at the femoral shaft.

Five synthetic distal femora were produced from resin (SL170; 3D Systems Europe Ltd., Hemel Hempstead, UK) using a stereolithographic process. The geometry of the cortex was designed to reproduce the anatomy of the distal femur and to fit a size-3 femoral component of a PFC TKA (Press-Fit Condylar; Johnson and Johnson, Raynham, MA). The cured resin had an elastic modulus of 3.7–4.2 GPa, approximately one quarter of the stiffness of healthy femoral cortical bone. There was a standardized F2b defect according to the AORI classification (Engh and Ammeen 1999) of approximately 10 cm^3^ at each condyle (Figure 1). Preliminary testing and evaluation was carried out to develop specific instrumentation and a standardized technique to ensure reproducible, consistent impaction of the morselized grafts.

**Figure 2.**
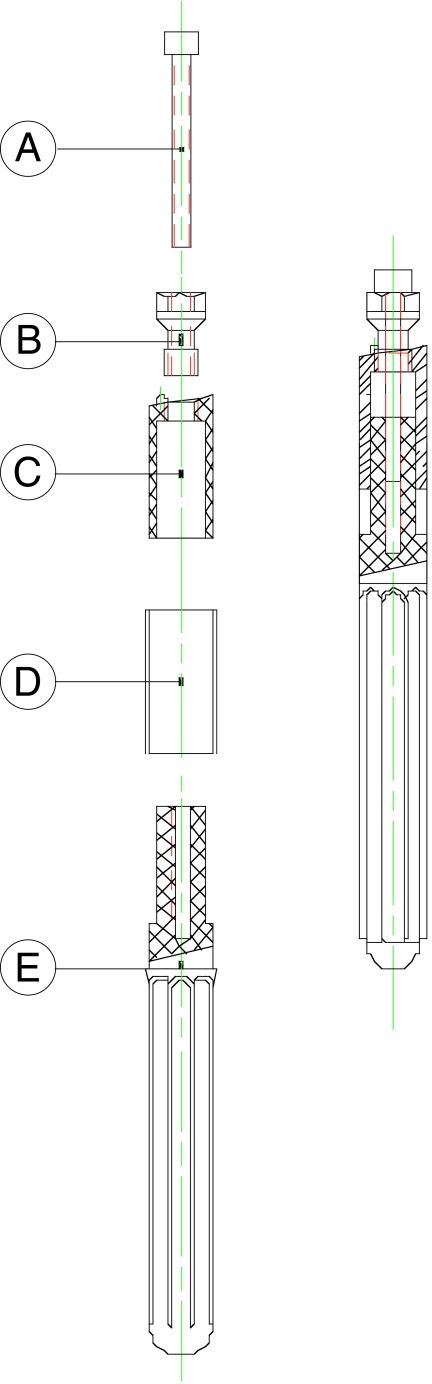
The sliding stem mechanism. A. Inner screw to lock or initiate the sliding mechanism. B. Hexagonal screw to connect the stem to the intercondylar box of the femoral component. C. Sliding part of the stem. D. Cylindrical protector, keeping the bone graft out of the sliding mechanism. E. Fluted stem with inner diameter of 10 mm and outer diameter of 12 mm.

The synthetic distal femur was clamped in a holder in an upside-down position. A guide wire was screwed at the bottom, centrally in the intramedullary canal and 170 mm proximal to the most distal point of the femoral cortical shell. Fresh-frozen femoral (bovine) heads were morselized using a bone mill (Noviomagnus; SMT, Nijmegen, the Netherlands). Two different cutters produced bone particles with a diameter of approximately 2.0 mm and 6.0 mm (Bolder et al. 2003). The small grafts were used for the shaft and the larger grafts were used to reconstruct the metaphysis and the condyles. The morselized grafts were impacted in the diaphysis in a stepwise manner, sliding over the guide wire with different tapered impactors, similarly to the femoral reconstructions in revision THAs (Schreurs et al. 1994). The final impactor had the same diameter as the later-inserted fluted stem; the sliding mechanism had a diameter of 15 mm and the fluted part had an inner diameter of 10 mm (Figure 2). The additional wings of the fluted stem generated further compression of the bone graft. The smallest inner diameter of the femoral shaft was 21 mm. Thus, we theoretically created a circumferential layer of IBG around the impactor of at least 3 mm.

The final impactor was kept in the femur during building of the metaphysis and the condyles. We used a template to contain the condylar defects during impaction. The template had a shape such that after firm impaction of the morselized grafts and subsequent removal of the template, the condyles were completely reconstructed and a revision prosthesis would fit exactly to the reconstructed bone. Bone cement (CMW3; CMW/De Puy, Blackpool, UK) was prepared and the guide wire, the final impactor, and the template were gently removed. A tantalum pellet with a diameter of 0.8 mm was glued to the tip of a 125-mm fluted stem.

A thin liquid cement layer was applied to the femoral component, whereas the stem and the intercondylar box were left free of cement and the component was cemented to the distal femur. There was no contact of the femoral component with any distal cortex, because the impacted reconstruction involved the whole distal femur. The stem was connected to the intercondylar box of the femoral component by a custom-made hexagonal fixation screw. A 6-Nm moment, measured with a torque wrench, was applied to the screw to provide a standardized connection of the stem to the intercondylar box. Into this screw, another screw was designed to lock or initiate the sliding mechanism of the box towards the stem (Figure 2). 5 specimens were prepared for mechanical testing.

### Mechanical testing

The reconstructed distal femur was clamped in the upside-down position in a testing machine (MTS model 458020; MTS Systems Corporation Minneapolis, MN) with the joint line parallel to the working bench. 6 tantalum pellets, diameter 0.8 mm, were glued with a connector to the femoral component and another 6 pellets were inserted in the shaft of the distal femur model (Figure 1). A unicondylar axial load cycling between zero and 500 N at 1 Hz frequency in series of 8 loading cycles was applied, alternating between the medially and laterally prosthetic condyles. Thus, we cyclically loaded the medially condyle 8 times and subsequently the laterally condyle 8 times. We chose this loading regime to rigorously assess the varus-valgus stability of the reconstruction.

**Figure 3.**
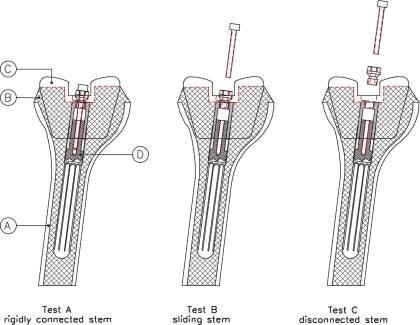
Schematic representation of tests A, B, and C. The distal femur is marked as “A”, the impacted bone graft as “B”, the femoral component as “C”, and the sliding stem connection as “D”. During test A, the femoral component was extended with a rigidly connected stem. Before test B, the inner screw was removed to initiate the sliding stem mechanism. To disconnect the stem from the femoral component, the hexagonal screw was removed before test C.

The loading tests were performed in the following sequence. Test A: 1800 cycles with a rigidly connected stem. Test B: 1800 cycles with a sliding stem. Test C: 1800 cycles with a disconnected stem. Between tests A and B, we removed the inner screw to initiate the sliding stem mechanism (Figure 3). Complete disconnection of the stem from the femoral component as tested in test C was achieved by removing the hexagonal fixation screw, whereas the stem remained in the intramedullary canal. All the experiments were performed by one surgeon (HJM). Stereoradiographs of unloaded and medially and laterally loaded situations were produced at the beginning and at the end of tests A, B, and C.

### Statistics

The stereoradiographs were digitized manually to determine the positions of the pellets and their 3-D positions were calculated using specialized software (Selvik 1989). The center of the intercondylar box was chosen as the origin of the coordinate system relative to which rotations and translations of the component in relation to the femur were expressed. Migration was calculated as 3 translations along and 3 rotations about the femoral axes. However, we focused the results on the translation of the prosthesis in axial direction and the prosthetic rotation in varus-valgus and flexion-extension directions. In an earlier knee kinematic study performed at the authors’ institution, the estimated error for the same RSA set-up was less than 50 μm for repeated measurements, with a standard deviation of 0.1 mm (Blankevoort et al. 1988). Statistical analysis of the dataset was performed with Friedman repeated measures analysis of variance by ranks, followed by Wilcoxon signed rank tests of differences in migrations between tests A and B and between tests B and C; p-values less than 0.05 were considered significant.

## Results

**Figure 4.**
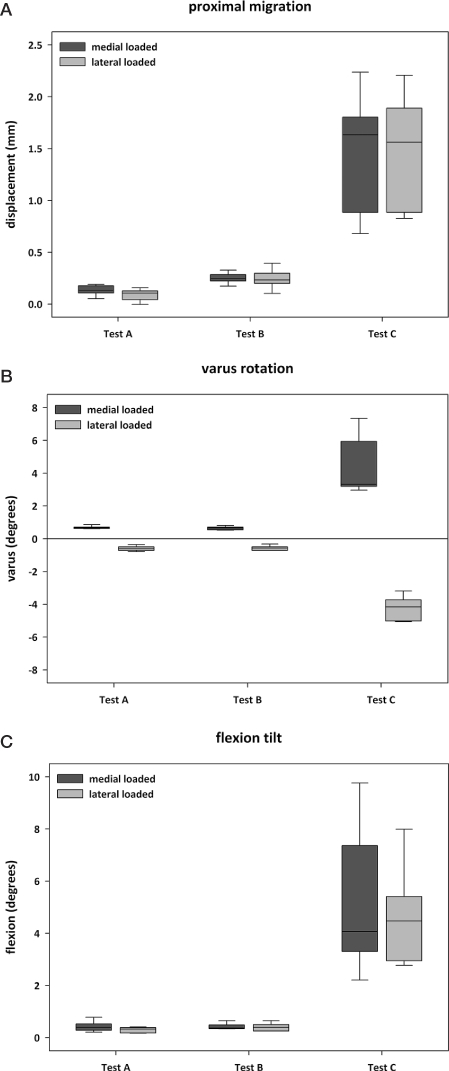
Box plots with medians and interquartile ranges of the proximal migration (panel A), varus rotation (panel B), and flexion tilt (panel C) of the femoral component after 30 minutes of loading during tests A, B, and C.

On visual inspection during the alternating medially and laterally axial loading, there was a stable reconstruction of the bicondylar femoral defects with IBG in the case of a rigid stem connection being used (test A). After 30 min of alternating axial loading, the stereoradiographs showed that the median proximal migration of the femoral component with a rigid stem connection was 0.13 (0.05–0.19) mm in the case of medially loading and 0.11 (0–0.16) mm in the case of laterally loading (Figure 4A). On the same stereoradiographs, the median varus rotations were 0.65 (0.61–0.86) degrees and –0.60 (–0.36 to –0.79) degrees, respectively, and the median flexion tilt 0.40 (0.21–0.78) degrees and 0.33 (0.17–0.41) degrees, respectively (Figure 4B and C).

After changing the stem connection from rigid to sliding, the reconstruction did not produce much more rotational migration than with the rigid connection (Figure 4B and C); there were no statistically significant differences in any rotational migration observed between the reconstruction with a rigid stem connection and the reconstruction with a sliding stem connection (Table). However, the sliding stem allowed proximal migration of the condylar component onto the femoral condyles, thereby compressing the impacted bone grafts. The average increase in proximal migration after 30 min of loading with the sliding stem compared with the loaded rigid stem connection was 0.14 mm.

**Table:** P-values of the differences in proximal migration, varus rotation, and flexion tilt during medially and laterally loaded situations after 30 minutes of alternating axial loading

	Test A versus B		Test B versus C	
	Medially loaded	Laterally loaded	Medially loaded	Laterally loaded
Proximal migration	0.04	0.04	0.04	0.04
Varus rotation	0.2	0.3	0.04	0.04
Flexion tilt	0.9	0.1	0.04	0.04

After complete disconnection of the stem, the reconstructions showed a high degree of instability with extrusion of the impacted graft under the component. There were statistically significantly more rotational and translational migrations of the femoral component in the reconstruction without a functional stem extension (p = 0.04) (Table). The stereoradiographs of the loaded situations showed that the median proximal migrations of the femoral component without a functional stem connection after 30 min of alternating axial loading were about 1.5 mm (Figure 4A). At the same time, the median varus rotations were more than 3 degrees, and the median flexion tilts were about 4 degrees (Figure 4B and C).

## Discussion

There have only been a few reports of genuine impaction bone grafting in revision TKA (Ullmark and Hovelius 1996, Heyligers et al. 2001, Lonner et al. 2002, Lotke et al. 2006, Steens et al. 2008). Ullmark and Hovelius (1996) published the first description of the technique. They essentially adopted the Slooff-Ling hip concept of a short stem totally surrounded by graft and cemented in situ (Gie et al. 1993). Although the early clinical results of IBG in revision TKA have been promising (Ullmark and Hovelius 1996, Bradley 2000, Heyligers et al. 2001, Lonner et al. 2002, Lotke et al. 2006, Steens et al. 2008), the mechanical stability of the reconstruction of bicondylar defects with IBG has not been described. Our study shows that a stable reconstruction of uncontained bicondylar femoral defects could be created with IBG and a TKA with a thin stem extension.

Although IBG is time consuming and technically demanding as regards incorporation of the bone graft into host bone and remodeling over time (van Loon et al. 2000a, b, Ullmark and Obrant 2002), IBG has excellent durability and versatility. Thus, compared to reconstructions with cement or metal augmentations, the restoration of the bone stock with IBG is preferable, particularly in younger patients if a further revision in future is considered likely.

It appeared that the presence of a functional stem extension was important for the stability of the bicondylar reconstruction. After disconnection of the stem, the femoral component showed more rotational and translational migrations with visible extrusion of the graft under the component. Previous reports have already suggested the necessity of a stem extension in revision TKA with bone grafting (Bourne and Finlay 1986, Ewald 1989, Ghazavi et al. 1997, Engh and Ammeen 1999, Nelson et al. 2003). Moreover, an earlier study at the authors’ institution on the reconstruction of unicondylar femoral bone defects had already demonstrated that a stem extension of the femoral component in TKA increases mechanical stability (van Loon et al. 2000c). In that study, bone grafting provided only a minor contribution to stability compared to a stem extension.

Despite these advantages, the disadvantage of a femoral component extended with a rigid stem is that long-term bone resorption is promoted due to stress shielding (Brooks et al. 1984, Bourne and Finlay 1986, van Lenthe et al. 1997, 2002). Hence, an incompatibility is present, which has prompted an ongoing discussion in the recent literature on the best way of stem fixation (Nelson et al. 2003, Mabry and Hanssen 2007, Wood et al. 2009). Although the use of cementless stems is currently more popular, the available literature suggests that cemented stem fixation provides a more reliable and durable construct for revision TKA associated with severe bone deficiency (Fehring et al. 2003, Whaley et al. 2003, Mabry and Hanssen 2007). Nevertheless, an FE study of femoral stems in revision TKA showed that cemented stems reduced more than half of the load transferred to bone graft under the femoral component, while press-fit stems reduced it only by one-sixth relative to stemless implants (Completo et al. 2009). The authors concluded that the higher levels of load reduction can promote late resorption of the graft and they advocated press-fit stems as a more adequate choice after graft incorporation.

Based on the fact that previous FE models of TKAs predicted less bone resorption when the femoral reconstruction was less rigid or fully unbonded (van Lenthe et al. 2002), we developed a sliding stem mechanism. In the present study, any rotational migration was similar between the reconstruction with a rigid stem connection and the reconstruction with a sliding stem connection. However, the sliding stem allowed proximal migration of the condylar component onto the femoral condyles, thereby compressing the impacted bone grafts. This supports our hypothesis concerning the sliding stem mechanism that adequate stability is provided by the sliding stem, while compressive contact forces are still transmitted to the distal femoral bone. Clinical studies will have to confirm that our sliding stem mechanism reduces stress shielding and maintains bone quality after revision TKAs.

The question remains as to how much stem sliding is acceptable. In this study, the sliding stem showed no more than 0.20 mm of shortening. The extent of sliding probably depends on the quality of the IBG at the femoral condyles. With continued loading, the condylar component further compresses the bone graft in the case of less firmly impacted reconstructions. The worse the quality of the IBG, the more proximal is the migration of the condylar component. Proximal migration of the joint line produces ligamentous instability in extension and causes impaired functional results of a TKA (Parratte and Pagnano 2008, Sikorski 2008). Thus, the sliding stem mechanism will only be successful if combined with a proper impaction technique.

As in all experimental studies, the present study had some shortcomings. First, the synthetic distal femora had an elastic modulus of approximately one quarter of the stiffness of healthy femoral cortical bone, which should therefore be regarded as a worst case of osteoporotic bone, as often occurs in revision TKA with IBG. The advantage of using artificial bones was that the geometry of the 5 specimens was exactly the same, which optimized the reproducibility of the results obtained in the tests. Secondly, we did not use a highly standardized impaction technique, for example, by using dropping weights as is used in other studies. We selected a more clinically relevant impaction technique (stepwise impaction by a single surgeon) because the impaction in the condylar area was done from all kinds of angles in order to obtain a firm and stable bone construct. Moreover, the reconstructed specimens served as their own control, which made the outcome less sensitive to variations in impaction grade between the specimens. Thirdly, the alternating loading of 500 N seemed to be low. Although the distal femur is normally loaded up to 2.5 times body weight during walking (Taylor et al. 1998), both femoral condyles share the patient’s body weight and a delay in full weight bearing is commonly advised when bone grafting is performed. Thus, the unicondylar load with 500 N in our study exceeded the clinical situation directly after surgery. Furthermore, with the alternating way of loading of the medially and laterally condyles, our study design was somewhat unconventional. However, from this the (varus-valgus) stability of the reconstruction was tested in a much more rigorous way than if we had used a dynamic force at a constant point of application. Fourthly, a potential danger of sequentially testing of a rigid stem, a sliding stem, and a disconnected stem is that earlier tests will influence the later tests because of accumulated damage. However, the results of the rigid stem connection and the sliding stem connection did not show any progressive migration during the 30 min of loading; the minimal migrations in the case of a rigid and a sliding stem were negligible compared to the migrations we found after disconnection of the stem. Thus, it is unlikely that considerable damage had accumulated in the constructs with a rigid or sliding stem and this indicates that those earlier tests did not influence (or only very mildly influenced) the high degree of migration in the case of a disconnected stem. Moreover, the advantage of sequential testing with one reconstructed specimen is that the reconstruction serves as their own control, with the type of stem fixation as the only variable. Fifthly, the standardized created defects of the femoral condyles had a flat surface, whereas in clinical practice these defects are usually irregular. Finally, the stem was not removed from the intramedullary canal after disconnection, to avoid removal and reapplication of the cemented femoral component. The presence of the stem in the canal after disconnection may have influenced the varus and valgus bending of the distal femur on loading, but probably did not influence the movements between the femoral component and the distal femur.

In summary, the present study shows that a stable reconstruction of uncontained bicondylar femoral defects could be created with IBG and a TKA with a thin stem extension. It appears that the presence of a functional stem extension is important for the stability of the bicondylar reconstruction. In an effort to reduce stress shielding, we developed a sliding stem mechanism. This sliding stem provides adequate stability, while compressive contact forces are still transmitted to the distal femoral bone. Clinical studies must still confirm that our sliding stem mechanism leads to long-term bone maintenance after revision TKAs.
